# An Overview of Brucellosis in Cattle and Humans, and its Serological and Molecular Diagnosis in Control Strategies

**DOI:** 10.3390/tropicalmed3020065

**Published:** 2018-06-14

**Authors:** Muhammad Zahoor Khan, Muhammad Zahoor

**Affiliations:** 1 Key Laboratory of Agricultural Animal Genetics and Breeding, National Engineering Laboratory for Animal Breeding, College of Animal Science and Technology, China Agricultural University, Beijing 100193, China; zahoorkhattak91@yahoo.com; 2 Department of Molecular Medicine, Institute of Basic Medical Sciences, University of Oslo, Sognsvannsveien, 90372 Oslo, Norway

**Keywords:** brucellosis, cattle, human, serological and molecular methods

## Abstract

Brucellosis is one of the most common contagious and communicable zoonotic diseases with high rates of morbidity and lifetime sterility. There has been a momentous increase over the recent years in intra/interspecific infection rates, due to poor management and limited resources, especially in developing countries. Abortion in the last trimester is a predominant sign, followed by reduced milk yield and high temperature in cattle, while in humans it is characterized by undulant fever, general malaise, and arthritis. While the clinical picture of brucellosis in humans and cattle is not clear and often misleading with the classical serological diagnosis, efforts have been made to overcome the limitations of current serological assays through the development of PCR-based diagnosis. Due to its complex nature, brucellosis remains a serious threat to public health and livestock in developing countries. In this review, we summarized the recent literature, significant advancements, and challenges in the treatment and vaccination against brucellosis, with a special focus on developing countries.

## 1. Introduction

Brucellosis is thought to have been identified in the late Roman era, named because of its resemblance to the organism *Brucellae* (later called *Brucella*) from carbonized cheese. Brucellosis has been associated with military campaigns, predominantly in the Mediterranean region. The disease was first expounded by Sir David Bruce, Hughes, and Zammit while working in Malta; hence the name ‘Malta fever’ is occasionally used for typical fever conditions caused by *Brucella* and its two most common species *B. abortus* and *B. melitensis*. *B. abortus* was first reported as a causative agent of premature delivery in cattle and intermittent fever in humans [1,2]. Brucellosis stands first in the list of zoonotic bacterial diseases, and 500,000 cases are reported annually in disease-endemic regions [3,4,5,6,7].

**Figure 1:**
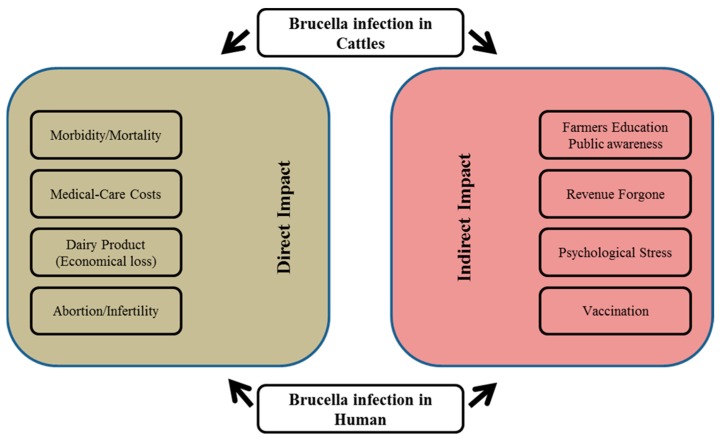
Summarizing the impact of *Brucella* infection in humans as well in cattle.

Although brucellosis is a widespread livestock infection in the Middle East and North Africa, it has not been studied in detail, except for rough figures about the epidemiology of the infection in these regions [8]. The bacteria infect reproductive tissues, lymph nodes, and the spleen, and therefore cause inflammation, edema, and necrosis. In pregnant animals it causes placental lesions and increases the risks of abortion [9,10]. Brucellosis gains public health importance when the bacteria are transmitted to human via unpasteurized milk, meat, and animal byproducts, from infected animals [11]. Proper diagnosis is one of the key obstacles for the complete eradication of brucellosis. Although several serological tests such as the Rose Bengal tube test, serum agglutination test, and enzyme-linked immunosorbent assay (ELISA) are used for disease diagnosis in cattle; however, these are often found to be misleading [12]. In recent years, PCR-based validation along with serological tests are widely used to ensure proper diagnoses [13]. Apart from the risk to public health, it also raises financial concerns to livestock stakeholders or latent product consumers. Figure 1 is a graphical summarization of brucellosis infection [14,15]. 

### 1.1. Brucella: The Causative Agent of Brucellosis

Brucellosis is caused by *Brucella*, a Gram-negative, aerobic, and facultative intracellular coccobacillus [16]. Based on taxonomic distribution, *Brucella* is classified as α-proteobacteria, which is further divided into six species, each including several biovars. The species *B. melitensis* biovars 1–3 have been reported in sheep and goats, and *B. abortus* biovars 1–6 and 9 in cattle. Similarly, the *B. suis* biovars 1–3 are known to infect pigs, while *B. suis* biovar 4 and 5 are more common for infection in reindeer and small rodents. Among other common species, *B. canis is* found in dogs, *B. ovis* in sheep, and *B. neotomae* in desert wood rats. Recently, *B. pinnipedialis* (in seals) and *B. ceti* (in whales and dolphins) are newly reported species, infecting marine animals [17].

The genome structure of *Brucella* is composed of two chromosomes, without plasmids, making it unique in Bacteriaceae. The recent introduction of genome sequence projects and genome information of *B. melitensis* (Gene Bank NC003317) and (NC003318), *B. suis* (Gene Bank NC002969), and *B. abortus* has opened up further gates towards the understanding of the disease pathogenicity and its mode of virulence [18,19]. Classification is usually based on the distinction between pathogenicity and host partiality [20]. *B. abortus* and *B. melitensis* are the key bovine brucellosis bacteria, while *B. abortus*, *B. melitensis*, *B. suis,* and *B. canis* are known for their infectivity in humans. Studies have also reported *B. melitensis* infection in sheep and goats [21,22].

### 1.2. Brucellosis Transmission

The infection of *Brucella* species is commonly mediated by direct contact with the placenta, fetus, fetal fluids, and vaginal discharges or byproducts (e.g., milk, meat, and cheese) from infected animals [23,24]. This explains why the typical route of infection is either direct ingestion or via mucous membranes, broken skin, and in rare cases intact skin [25,26]. Professional health workers are frequent victims of *Brucella* infection, especially in regions of prevalent disease, and it is documented that nearly 12% of laboratory workers in Spain get brucellosis during fieldwork [27,28]. In addition, in utero transmission, person-to-person transmission, and transmission associated with tissue transplantation have been observed in rare cases [29,30,31]. Aerial bacteria also remain a severe threat of infection, either by inhaling organisms or through the conjunctiva. Brucellosis also spreads via vertical transmission, by infecting new-born calves and lambs in the uterus [32].

### 1.3. Global Public Health Concerns

Brucellosis has been reported in 86 different countries worldwide and is a serious threat not only to livestock but also to human health globally. Despite its brutal impact on economic loss, it is also associated with high morbidity, both for humans and animals in developing countries [25,33]. North African and Near East countries are listed at the top for infection and cross-infection of brucellosis [34,35]. *Brucella melitensis* and *B. abortus* persistence has been confirmed in most Middle Eastern countries, but African and Asian continents are not spared either [36,37]. *Brucella abortus* and *B. suis* infection is widespread throughout Central America [38]. In Europe, human brucellosis is thought to be associated with travellers and immigrants from the Middle East or the private import of dairy products from endemic areas [37,39,40,41].

*Brucella* infection is widespread in several South Asian/Asian countries including Pakistan, India, China, and Sri Lanka, in humans as well as in animals [42,43,44,45]. In 1950, *Brucella* was for the first time reported in animals in Malaysia, and the government undertook an eradication strategy for bovine, ovine, and caprine brucellosis (National Surveillance Program for Animal Brucellosis) since 1978 [46]. Additionally, a series of studies documented the seropositive cases of brucellosis in humans mainly in veterinary professionals and farmers that had close contact with animals. The prevalence of brucellosis is more common in males (90%) ranging from 20–45 years old in Malaysia [47]. This showed that *Brucella* infection is highly zoonotic, as males are commonly involved in the handling of livestock and their products in Malaysia. Brucellosis occurrence fluctuates extensively, not only between countries but also within a country.

Though we lack solid evidence, a report suggests that in Iraq and Egypt occupation and socioeconomic status are associated with the rate of *Brucella* infection [35,48]. This possibly explains the high brucellosis incidence in low- and middle-income countries. To further endorse this, it was not surprising that brucellosis is more common in specific communities even in developed countries, such as Turkish immigrants in Germany or Hispanics in the USA—communities with poor socioeconomic status [49,50]. The studies above are enough to assume that though brucellosis is common in underdeveloped/developing countries or even in communities with poor socioeconomic status, in developed countries due to its infectious nature, the risk circle of *Brucella* infection might potentially extend to safe havens in the near future [51,52].

Dissecting the occupational hazard of brucellosis, the disease is commonly found in shepherds, people working in the dairy or meat industry, veterinarians, and laboratory professionals. Males are more prone to infection compared to females, being more likely to adopt such occupations. However, in rural areas where women handle livestock, the incidence rate is elevated in females [53,54]. Brucellosis prevalence is common in people of the age group 13–40 years; in northern Saudi Arabia, it decreases in the older aged group [55]. However, vulnerability gets worse in aged groups, and can even lead to destructive localized brucellosis of the spine in cases of acute localized brucellosis [56]. Children are rarely susceptible to brucellosis, except in the regions that lack the proper pasteurization of milk [57]. This leads us to conclude that brucellosis does not associate with gender and age, but rather occupation and exposure to bacterial infection.

### 1.4. Clinical Picture of Brucellosis in Cattle

Brucellosis is a widespread reproductive disease, commonly causing abortion, death of young ones, stillbirth, retained placenta or birth of weak calves, delayed calving, male infertility, and marked reduction in milk yield [37,58,59,60]. It infects almost all domestic species except cats, which are naturally resistant to *Brucella* infection [59]. In bulls, the disease is characterized by fever, vesiculitis, orchitis, and epididymitis. In severe cases, it can also be the reason for testicular abscesses, metritis or orchitis that can lead to lifetime infertility. In animals, brucellosis symptoms can be varied from severe acute to sub-acute or chronic, depending upon the organ of infection and the type of animal [60]. When a pregnant animal is infected by *Brucella*, a visible swelling of the mammary gland to the navel region and bleeding from the vagina is not uncommon, even if the cow does not abort. The enlarged udder size (appearance of the 9th month of a pregnant cow) could be used as an indication for the high stage of the disease, where animals shed bacteria in urine, milk, and vaginal discharges.

### 1.5. Human Brucellosis

Human brucellosis is known by many different names such as Malta fever, Cyprus or Mediterranean fever, intermittent typhoid, rock fever of Gibraltar, and more commonly, undulant fever [61]. The usual incubation period of one to four weeks can be extended up to several months before complete symptoms appear. Infection among children is generally more benign than in adults, concerning the likelihood and severity of complications and response to treatment [62]. 

Fever is one of the most common symptoms across patients; intermittent in 60% of patients with acute and chronic brucellosis, while undulant in 40% of patients with subacute brucellosis. Fever is thought to be linked to relative bradycardia and fever of unknown origin (FUO) is a more common initial diagnosis in patients in areas of low endemicity. Nearly 80% of patients suffer from chills, and 20% of patients develop a cough and dyspnea without any active pulmonary involvement. Additionally, pleuritic chest pain may affect patients with underlying empyema [16,63,64]. 

Brucellosis also increases the risk of spontaneous abortion, premature delivery, miscarriage, and intrauterine infection with fetal death in humans as well, which is accompanied with malaise, fatigue, and arthritis [28,63]. Septicemias with sudden onset followed by high fever, emaciation, restlessness, undulant fever, sexual impotence, insomnia, headache, loss of appetite, and weight loss can also be seen in an infected patient [65]. The detailed symptoms of brucellosis have been documented; however, due to their protean and complex nature, clinical manifestations cannot be relied on for diagnosis [66]. In humans, brucellosis is not confined to the reproductive system, but is also known to cause neurobrucellosis with clinical manifestation of meningitis, encephalitis, stroke, radiculitis, myelitis, peripheral neuropathies, and neuropsychiatric features [67,68]. Studies have also reported sensorineural deafness, spastic paraparesis, followed by brisk tendon reflexes, bilateral ankle clonus, and extensor plantar responses [69].

## 2. Diagnosis of Brucellosis

### 2.1. Serological Tests

At the moment, no specific diagnostic test is available to identify *Brucella.* Therefore, conventional serological examination must be accompanied with more supportive analysis [59,70]. Serological methods are used for the initial screening of human brucellosis, as well as during follow-up treatment. Due to the consistent false negativity of serological tests in early days of infection, serial serological testing is usually recommended, which will not only help in proper diagnosis but also add to monitoring for response to treatment. 

During the first week of illness, the changes in immunoglobulin (Ig) M isotype antibodies predominate, followed by an elevated level of IgG in the second week [71]. The titers of both subtypes continuously increase and reaches the peak within four weeks. Generally, a decline in antibody levels can be seen after antibiotic treatment, while relapse is often characterized by a second peak of anti-*Brucella* IgG and IgA, but not IgM [72]. At present, no standardized reference antigen for serological tests is available, therefore, combinations of several serological tests are recommended.

The investigative antigen of standard serological tests is usually prepared from whole-cell extract, which is majorly constituted of smooth lipopolysaccharides (S-LPS). During natural infection, the humoral immune reaction is characterized by antibody production against S-LPS, and therefore, diagnostic assays identify agglutinating and non-agglutinating antibodies. However, the diagnostic tool based on anti-LPS detection might lose its specificity due to its cross-reactivity with other clinically-relevant bacteria.

The immune-dominant epitope of the *Brucella* O-polysaccharide shows similarities with many other bacteria, such as *Yersinia enterocolitica* O:9, *Salmonella urbana* group N, *Vibrio cholerae*, *Francisella tularensis*, *Escherichia coli* O157, and *Stenotrophomonas maltophilia* [72,73]. Some *Brucella* species do not share similarities in S-LPS antigen, due to which the current conventional serological test loses its global application. Canine brucellosis, caused by *B. canis*, lacks S-LPS antigen, and thus cannot be diagnosed by standard S-LPS-based serological assays [74].

Among the serological methods currently in practice, the serum agglutination test (SAT) is commonly used for the diagnosis of *Brucella* infection in humans [72]. The updated serum tests (slide, plate, and card agglutination) have replaced the laborious and time consuming methods (i.e., Wright test) that were routinely used for clinical diagnosis of brucellosis. The Rose Bengal test (RBT) is an example of a card test used in endemic countries for the rapid diagnosis and screening of patients in emergency departments [75]. However, it is generally recommended that the RBT must be used in combination with other standard serological tests for more reliable detection and to avoid false positives. In high-risk populations, testing of diluted sera using the RBT might be a reasonable choice to reduce the need for a huge number of assenting tests [76]. The significance of diagnostic titers in follow-up sera from patients with brucellosis can be examined only within the circumstance of a well-matched clinical representation [69]. The lateral flow assay is another tool appropriate for rapid field or bedside testing in low socio-economic endemic areas, where laboratories lack modern facilities. This assay is even considered more accurate and specific than the SAT in chronic and complex cases [77]. 

Acomparative analysis of three tests (RBT, SAT, and Coombs’ test (CT)) recommended Coombs gel test regarding specificity and sensitivity [78]. Several other serological tests are also used for diagnosis including the standard tube agglutination test (STAT), enzyme-linked immunosorbent assay (ELISA), milk ring test (MRT), and fluorescence polarization assay (FPA) [79]. Among them the SAT remained the most popular and used test for routine diagnostic practice worldwide [49]. Immunoglobulins including immunoglobulin M (IgM), IgG, and IgA measurement by ELISA reflect the better image of clinical disease manifestation. Compared to the SAT, ELISA yields higher sensitivity and specificity, therefore it is widely used in the diagnosis of chronic cases of brucellosis to detect incomplete antibodies [80,81].

Complement fixation test (CFT) is an option developed for the detection of IgG, but mostly used as a confirmatory test because of its cross-reactivity with *B. abortus* S19 vaccinated cattle [82]. The classical CT helps in the detection of incomplete, non-agglutinating or blocking antibodies, and is considered a suitable test to detect slight changes in anti-*Brucella* antibody titers during relapse and chronic courses [73]. 

Despite the fact that several serological assays are available in clinics, none of them meet the standard criteria for a convincing diagnosis. None of the assays are recommended to be used alone in endemic areas, and a verification test is often required [83].

Due to the lack of specificity and sensitivity of serological tests and culture techniques, different molecular methods have been optimized both for the diagnosis of bovine and human brucellosis [84].

### 2.2. Molecular Diagnosis

Polymerase chain reaction (PCR)-based diagnosis has been adopted in recent decades and is rapidly replacing conventional assays for diagnosis in clinical laboratories. In the same fashion, PCR-based detection of *Brucella* has also emerged as a novel and much more efficient diagnostic tool. Moreover, it not only detects but also accurately distinguishes between acute, subacute, and chronic infection. The pioneering approach using PCR for *Brucella* diagnosis was reported in early 1990s [85]. Blood is an easy source of DNA for the diagnosis of *Brucella* infection. In addition, various other clinical specimens including serum, urine, and cerebrospinal, synovial or pleural fluid and pus can also be used for *Brucella* detection [86,87]. In recent years, serum is the preferred source of DNA in molecular diagnostic assays, due to its anticoagulant and hemoglobin-free nature.

The detection of *Brucella* DNA in patients is considered a challenging task because of the lower number of bacteria in infected tissue and the inhibitory effects taking place from surrounding substances [88]. The standard methods used for sample preparation must include a step that reduces matrix inhibitory influences and deliberate bacterial DNA. Additionally, the residual PCR inhibition by complex matrices can also be overcome through the use of proper internal amplification control [89]. The QIAamp™ DNA Mini Kit (Qiagen Inc., Valencia, CA, USA) and the UltraClean™ DNA Blood Spin Kit (MO BIO Laboratories Inc., Carlsbad, CA, USA) are commercially-available kits, ready to be used for *Brucella* DNA extraction from serum, blood, and other tissue samples [90]. The circulating macrophages engulf and processes bacteria and negatively affect the PCR-based detection. However, the modern PCR method has the ability to detect even the non-viable or phagocytosed microorganisms [91]. *Brucella* DNA has also been successfully detected in milk samples from an infected animal using PCR-based assay [92]. 

Various gene and loci have been identified as potential targets for PCR-based amplification [50,93]. For example, *IS711* insertion element is a potential target that can be used for the detection of traceable bacteria as its multiple copies are found in the *Brucella* chromosomes [94]. Moreover, 16S rRNA also serves as a potential target, not only for *Brucella* but also related microorganisms [95]. The species-specific real-time PCR and conventional Bruce-ladder PCR assays are also considered to be key tools, used for confirmation and delineation of *Brucella* species [96]. For the diagnosis of human brucellosis, multilocus variable number tandem repeat analysis 16 loci panel (MLVA-16) is considered to be an authentic target [97].

Summarizing the facts, molecular diagnostics have the edge over conventional methods as they are robust and versatile, and due to the non-infectious nature of DNA, therefore safer for laboratory personnel. PCR-based detection is also more reliable and specific when compared to the serum plate agglutination test (SPAT) [98,99]. However, for a PCR-based assay, a specialized machine like a conventional thermocycler or real-time PCR is required along with skilled personnel. Moreover, specific primers for each *Brucella* species will be required.

## 3. Treatment of Brucellosis

Though the complex nature of brucellosis makes it harder to treat, long-term treatment with an antibiotic is thought to be beneficial. In most cases, antibiotics in combination are found to be more effective against the infection; however, the state of the disease still does not lose its importance [100,101]. Several conventional antibiotics including tetracycline, trimethoprim-sulfamethoxazole, aminoglycosides, rifampicin, quinolones, chloramphenicol, doxycycline, and streptomycin are commonly used in clinics [102,103]. In several cases, the application of antibiotics in a specific order has given best results. Likewise, a case reported that treatment with doxycycline for six months, followed by streptomycin for three weeks was found very effective against brucellosis in human [104]. Another study reported that the alkaloid columbamine in combination with jatrorrhizine were more effective against brucellosis caused by *B. abortus* compared to a combination of streptomycin and rifampicin [105]. The World Health Organization recommends that acute brucellosis cases be treated with oral doxycycline and rifampicin (600 mg for six weeks) [106]. However, rifampicin monotherapy is in common practice for treating brucellosis in pregnant women, and a combined therapy of sulphamethoxazole and trimethoprim is recommended for children [107]. In underdeveloped countries, treatment of cattle is not a common practice; however, the infected animals are isolated, culled or slaughtered to prevent the spreading of infection to other herd and at substantial veterinary costs. 

In China, a case of subdural empyema complicated by intracerebral abscess due to *Brucella* infection was effectively treated with antibiotic therapy (ceftriaxone, doxycycline, rifapentine) [108]. In line with this, several reports suggested the combination therapy of doxycycline and rifampicin for six weeks is enough to eradicate *Brucella* infection, as well as associated complications [46,109,110,111]. This combination of doxycycline and rifampicin has also been proven experimentally [112]. As a result of continued efforts by the scientific community to develop an effective therapeutics, *Caryopteris mongolica* Bunge (Lamiaceae) has been tested in combination with doxycycline [113,114]. Despite the fact that several therapeutics are in practice which makes the disease manageable, an effective therapeutic is required for the complete treatment of brucellosis.

## 4. Vaccination against Brucellosis

To overcome the widespread intra- and inter-species infection of brucellosis, potent vaccination would be the best strategy [115]. Currently, several vaccines including S19, RB51, *B. melitensis* Rev.1, lysate, live vectored vaccine, mucosal vaccine subunit, and DNA vaccines are available for brucellosis [116,117,118]. In cattle, *B. abortus* strain 19 and RB 51 are the most commonly practiced vaccines [119,120]. S19 is used to vaccinate young female calves (3 to 12 months); however, it is not recommended for pregnant cattle, as it results in abortion [121]. S19 was found more effective in developing long-term immunity, when compared with RB51, in young calves [116,122,123]. However, RB51 does not interfere with serological diagnosis [124,125].

S19 and RB51 are live attenuated vaccines derived from *B. abortus* [126]. A cocktail lysate of S19 and RB51 was also tested as an immune-therapy to treat the bracelet infected cattle [114]. DNA vaccines have also been tested and show promising results when compared with S19 and RB51; however, several boosters were required to achieve the desired immunity [127,128]. In China, the S2 vaccine is widely in practice; however, it triggers an innate immune response and causes increased inflammation [129]. In conclusion, no effective and relatively safe vaccine is available that provides long-term protection against brucellosis.

## 5. Control Strategies for Prevention of Brucellosis

An effective approach should be adopted to eradicate and prevent brucellosis in cattle and humans. Diagnosing, curing/eradicating, and prevention are the golden rules often recommended by experts [130,131]. The slaughtering and proper disposal of seropositive animals to decrease the incidence of infection in healthy animals and effective vaccination and hygienic practices would reduce the disease spreading in/from endemic regions [132]. Vaccination is an effective strategy to prevent the spread of brucellosis and is in practice worldwide. However, there is demand for the development of new vaccines that are safer and more effective [9].

To cover the zoonotic aspects of brucellosis, proper education of field farmers, field workers, and the local community in endemic regions is required. The effective pasteurization of milk and other products and disinfection of meat is of key importance before consumption. The regular sterilization of labwares and laboratory tools would also result in a decrease in infection of clinical laboratory personnel [133].

Apart from local efforts, an effective global policy is required for the complete eradication of brucellosis. Proper veterinary legislation must be implemented and policies regarding animal health need to be encouraged. Modern updated knowledge on brucellosis should be delivered to farmers, veterinary professionals, and health educators, especially for rural populations, which will help to prevail over the dispersal of *Brucella* infection [134,135]. 

## 6. Conclusions

Brucellosis is not only a threat to livestock but also a global public health issue. Unfortunately, we lack not only a proper treatment but also a reliable diagnosis. Adequate and timely diagnosis of brucellosis is necessary to control and treat the disease in the best way. Different serological and molecular methods are used for the screening of the disease. However, each test has some drawbacks in one way or another. So here we suggest that due to the zoonotic importance of the *Brucella* infection, it is necessary to handle the disease in a proper way and a combination of particular tests should be used to screen for brucellosis in both humans and animals. The different cited studies regarding brucellosis in humans and cattle revealed that the combination of both the molecular and serological methods must be practiced for accurate diagnosis. If the infected animals are in chronic infected condition, they should be culled to prevent the disease spreading. The formal education and necessary training of farmers, especially those living in rural areas, would also help to get control over the disease. With rising interest of the scientific community in brucellosis, a significant improvement in diagnosis and treatment is expected. We are also in need of a broad-spectrum vaccine against *Brucella* for complete eradication of the disease worldwide.
